# Remote Symptom Monitoring With Electronic Patient-Reported Outcomes in Clinical Cancer Populations

**DOI:** 10.1001/jamanetworkopen.2025.9852

**Published:** 2025-05-13

**Authors:** Gabrielle B. Rocque, Jeffrey A. Franks, Luqin Deng, Nicole E. Caston, Courtney P. Williams, Andres Azuero, D’Ambra Dent, Bradford E. Jackson, Chelsea McGowan, Nicole L. Henderson, Chao-Hui Sylvia Huang, Stacey Ingram, J. Nicholas Odom, Noon Eltoum, Bryan Weiner, Doris Howell, Angela M. Stover, Jennifer Young Pierce, Ethan M. Basch

**Affiliations:** 1 Department of Medicine, Division of Hematology and Oncology, University of Alabama at Birmingham, Birmingham; 2 Department of Medicine, Division of Gerontology, Geriatrics and Palliative Care, University of Alabama at Birmingham, Birmingham; 3 Department of Medicine, Division of General Internal Medicine and Population Science, University of Alabama at Birmingham, Birmingham; 4 O’Neal Comprehensive Cancer Center, Birmingham, Alabama; 5 School of Nursing, University of Alabama at Birmingham, Birmingham; 6 Lineberger Comprehensive Cancer Center, University of North Carolina at Chapel Hill, Chapel Hill; 7 Mitchell Cancer Institute, University of South Alabama, Mobile; 8 Department of Health Systems and Population Health, University of Washington, Seattle; 9 Supportive Care, Princess Margaret Cancer Centre Research Institute, Toronto, Ontario, Canada; 10 Department of Health Policy and Management, University of North Carolina at Chapel Hill, Chapel Hill

## Abstract

**Importance:**

Value-based health care increasingly requires electronic patient-reported outcome–based remote symptom monitoring (RSM) to improve health care utilization in patients with cancer. However, data on the impact of RSM in clinical practice are lacking.

**Objective:**

To evaluate the association of RSM with 3- and 6-month health care utilization among patients receiving systemic cancer treatment.

**Design, Setting, and Participants:**

This nonrandomized controlled trial used a hybrid, type 2 implementation-effectiveness design. Participants were patients with cancer at 2 Alabama-based academic institutions receiving chemotherapy, targeted therapy, or immunotherapy; the exposure group received standard-of-care delivered RSM from 2021 to 2024, and historical controls were patients who received cancer treatment prior to RSM implementation from 2017 to 2021. Data were analyzed from May to October 2024.

**Exposure:**

RSM using electronic patient-reported outcomes.

**Main Outcomes and Measures:**

Health care utilization at 3 and 6 months after RSM enrollment (intensive care unit [ICU] admissions, hospitalizations, emergency department [ED] visits). Adjusted modified Poisson models estimated the relative risk (RR) and 95% CI of health care utilization overall. Penalized logistic regression was used for stratified analyses by patient race, residence, neighborhood deprivation, insurance type, and comorbid conditions.

**Results:**

A total of 5949 patients were assessed. From May 2021 to May 2024, 1392 patients (median [IQR] age at index date, 61 [51-69] years; 933 [67%] female) were enrolled in RSM, including 378 Black patients (27%) and 922 White patients (66%), with 262 patients (19%) living in rural areas and 372 patients (27%) living in areas with high neighborhood disadvantage; RSM patients were compared with 4557 controls (median [IQR] age at index date, 62 [53-69] years; 2654 [58%] female), including 1177 Black patients (26%) and 3151 White patients (69%), with 1012 patients (22%) living in rural areas, and 1281 patients (28%) living in areas with high neighborhood disadvantage. Compared with historical controls, hospitalizations among patients receiving RSM were 19% lower at 3 months (RR, 0.81; 95% CI, 0.73-0.91) and 13% lower at 6 months (RR, 0.87; 95% CI, 0.80-0.96). ICU admissions were not significantly different among the RSM populations compared with controls (3 months: RR, 0.82; 95% CI, 0.59-1.13; 6 months: RR, 0.83; 95% CI, 0.65-1.06). ED visits were similar for both groups (3 months: RR, 1.02; 95% CI, 0.89-1.16; 6 months: RR, 1.03; 95% CI, 0.92-1.15). Subset analyses showed similar patterns in 3- and 6-month RR for hospitalizations, ED visits, and ICU admissions.

**Conclusions and Relevance:**

In this nonrandomized controlled trial, RSM implementation was associated with reduced risk of hospitalizations for patients with cancer, supporting the need to expand implementation nationally.

## Key Points

**Question:**

What is the association of electronic patient-reported outcome-based remote symptom monitoring (RSM) with health care utilization in patients with cancer?

**Findings:**

In this nonrandomized controlled trial including 5949 patients, with 1392 patients receiving RSM and 4557 historical controls, hospitalizations were 19% and 13% lower among patients receiving RSM at 3 and 6 months, respectively; however, emergency department visits and intensive care unit admissions were not significantly different between for patients receiving RSM vs historical controls.

**Meaning:**

This nonrandomized controlled trial found that RSM implementation was associated with reduced risk of hospitalizations for patients with cancer, supporting the need to expand implementation nationally.

## Teaser

This nonrandomized controlled trial evaluates the association of patient-reported outcome–based remote symptom monitoring with health care utilization among patients with cancer.

## Introduction

Value-based health care initiatives drive health care transformation. In 2016, the Center for Medicare & Medicaid Innovation (CMMI) launched the Oncology Care Model (OCM),^1^ a payment reform demonstration project. The OCM included key components of previously successful CMMI demonstration projects, such as the Patient Care Connect Program, highlighting benefits of lay navigation,^2^ and the COME HOME program, which reported cost savings from structured decision-support tools for both treatment and symptom management.^3^ Although the OCM integrated components of these projects that demonstrated value in oncology, the model had less value than desired. A lack of significant differences was observed in hospitalizations, emergency department (ED) visits, and survival between patients cared for in practices participating in OCM compared with those not participating. Furthermore, reductions in health care costs were offset by the up-front payments to practices, resulting in a net cost to Medicare.^4^ However, significant cost reductions of $503 per beneficiary per episode were identified among high-risk episodes, 5 quality metrics were improved, and the remainder of metrics remained similar to baseline, without any worsening.^4^ Furthermore, health care practices highlighted the benefits of OCM practice transformation on care delivery within their individual institutions.^5^ In addition, increased utilization of the practice transformation activities (eg, navigation, depression screening) was also seen in oncologic care nationwide, suggesting OCM policy changes may have influenced non-OCM practices to build infrastructure. Ultimately, this model was refined and relaunched as the new Enhancing Oncology Model (EOM) in 2024.^6^

While EOM differs from the OCM by several model characteristics, the practice transformation activities are consistent, with 1 key addition—the inclusion of electronic patient-reported outcomes (ePROs) to guide remote symptom monitoring (RSM). RSM includes delivery of electronic weekly symptom surveys, typically administered between clinic visits by email or text, with associated triggers for symptoms meeting a prespecified threshold to the clinical team to reach out and manage symptoms proactively. The choice to add ePROs for RSM is a natural fit for value-based health care because of both the potential to leverage underlying infrastructure built within OCM (eg, navigation, distress screening processes) and robust literature from randomized clinical trials that found ePRO-based RSM adds value. In trials conducted by Basch and colleagues,^7,8,9^ RSM improved symptom burden, quality of life, and time receiving treatment and reduced health care utilization. Specifically, in a trial of patients with advanced cancer, those receiving RSM had 7% fewer ED visits and 4% fewer hospitalizations than patients receiving standard oncology care and experienced a 5-month survival benefit.^8^ Similar national and international studies have further built the case for RSM using ePROs.^10,11,12^ As such, the oncology community has called for routine implementation of ePROs as part of standard care,^13^ a call now moved forward in EOM.^6^

Despite multiple successful clinical trials, these studies largely engaged small numbers of patients without full representation of patients encountered in routine clinical practice or at a scale expected for EOM. Furthermore, few practices have successfully implemented ePROs as a part of routine care. Despite implementation during the COVID-19 pandemic, a large community oncology practice demonstrated early implementation feasibility, engaging more than 4000 patients in their initial ePRO RSM demonstration, with 72% completing at least 1 ePRO assessment.^14^ Furthermore, reductions in hospitalizations (20% vs 33%) and ED visits (38% vs 42%) were observed for a subset of 229 Medicare patients receiving RSM matched to non–RSM-enrolled controls.^15^ While these results are promising, data are lacking on the impact in clinical settings outside the Medicare population and the impact on specific marginalized subpopulations (eg, Black patients, patients living in rural or highly disadvantaged areas) who face challenges with digital health literacy and use of technology within health care.^16^ These subpopulations are now engaging with ePRO-based RSM, with enrollment rates in diverse populations more than 90% and survey completions rates of 80%.^17^ However, given known racial, geographic, and socioeconomic disparities in health outcomes, it is imperative that health care delivery interventions also consider impacts in disparate populations to minimize risk of exacerbating disparities.

As practices consider the second invitation to join EOM and payers consider which practice transformations should be included within payment reform demonstrations, additional data on ePRO-based RSM are needed across diverse populations. Thus, this study evaluated the association of ePRO-based RSM delivered as part of standard care with health care utilization in a diverse health care delivery system caring for large proportions of patients who are Black, live in rural areas, or reside in communities with limited resources.

## Methods

This nonrandomized controlled trial was approved by the University of Alabama at Birmingham institutional review board with a waiver of consent for participants because the evaluation relied on secondary data. This study is reported following the Transparent Reporting of Evaluations With Nonrandomized Designs (TREND) reporting guideline.

### Study Design and Population

This study followed a nonrandomized, hybrid type 2 trial design^18^ to evaluate both implementation outcomes and effectiveness of ePRO-based RSM for patients with cancer (ClinicalTrials.gov identifier: NCT04809740). Implementation of this program occurred at the University of Alabama at Birmingham O’Neal Comprehensive Cancer Center and the University of South Alabama Mitchell Cancer Institute (MCI; located in Mobile, Alabama). These 2 academic medical centers serve a substantial population of patients who are Black, reside in rural areas, live in poverty, or who have low education levels. Design of the trial and analytic plans have been described previously.^18^ Implementation outcomes have been previously reported.^17,19,20,21,22,23^ This analysis focuses on the secondary outcome of health care utilization (ED visits, hospitalizations, ICU admissions) for patients receiving ePRO-based RSM.

### RSM Program

Patients with cancer eligible for RSM included patients at participating institutions receiving their first infusion or oral chemotherapy, immunotherapy, or targeted therapy. Prior cancer and associated treatment were allowed if it was administered at another institution. Patients receiving second opinions, who had a gap between enrollment and treatment start of more than 120 days, or without any survey completion were excluded. Programmatic details are outlined elsewhere.^17,19,20,24^ Briefly, nonclinical navigators identified eligible patients using electronic medical record (EMR) review and approached eligible patients in-person or via phone to enroll in a web-based ePRO reporting platform (Carevive; Health Catalyst) that was accessible on any smart device or computer. Patients could choose to decline enrollment. They then received weekly symptom assessment surveys to complete for 6 months, with responses integrated into the EMR. Surveys by patients who reported moderate or severe symptoms generated alerts to clinic nurses, who provided symptom management per institutional protocols. All clinicians were able to view ePRO data, which were integrated within the EMR. No incentives were provided, as this was a standard-of-care intervention.^9^

### Intervention and Historical Controls

Intervention patients were those enrolled into RSM from May 2021 to May 2024. Historical controls were identified in the EMR from 2017 to the time each disease team (eg, breast cancer, lung cancer) rolled out RSM. To compare intervention patients with controls, we used an index date, defined as the RSM start date for intervention patients or the date of the first RSM qualifying treatment for controls.

### Health Care Utilization

The primary outcomes for this analysis were hospitalization within 3 and 6 months of enrollment into the RSM program. Secondary outcomes included ED visits and intensive care unit (ICU) admissions at 3 and 6 months. If a patient was admitted to the hospital via the ED, then the patient was considered as having both an ED and hospital utilization. Health care utilization data were abstracted from the EMR, and each was dichotomized as a binary indicator.

### Variables

Patient data abstracted from the EMR included date of birth, sex (female, male), home address, insurance status (private, Medicaid, Medicare fee-for-service only, Medicare plus supplemental insurance, none/other), cancer type (breast, gastrointestinal, genitourinary, gynecological, head and neck, hematological [leukemia, lymphoma, myeloma], lung, melanoma, other), cancer treatment (chemotherapies, immunotherapies, and targeted therapies), and comorbidities using *International Statistical Classification of Diseases and Related Health Problems, Tenth Revision *(*ICD-10*) codes up to a year prior to the index date. Address data from the EMR were used to categorize patient rurality and neighborhood disadvantage. Rural-Urban Commuting Area Codes were used to assign patient residence as rural or urban using zip codes.^25^ The Area Deprivation Index, a percentile ranking measure from 1 to 100 that encompasses domains of education, income, employment, and housing quality at the neighborhood level, was used to determine patient neighborhood disadvantage using Census-block data. Area Deprivation Index scores were dichotomized, with neighborhoods percentile ranked from 86 to 100 considered more disadvantaged.^26^ The Elixhauser Comorbidity Index was used to quantify patient’s comorbidities and encompasses 31 groups where a larger score indicates greater comorbidities and projected hospital resource use.^27^ The ePRO data were abstracted from the web-based ePRO reporting platform. Other patient data abstracted from the ePRO platform included EMR-based race (Black, White, or other [eg, American Indian or Alaska Native, Asian, Hispanic or Latino, Native Hawaiian or Pacific Islander, other]). Races were collapsed due to sample size. Missing data from EMR abstraction of discrete fields were supplemented with EMR review and data from the ePRO platform. Only patients with complete data were included within the analysis.

### Statistical Analysis

Descriptive statistics were calculated using frequencies and percentages for categorical variables and medians and IQRs for continuous variables. Modified Poisson models with robust SEs^28^ were used to estimate the relative risk (RR) and 95% CI of any hospitalization, ED visits, or ICU use between patients receiving RSM and controls for 3 and 6 months after the enrollment or index date, respectively, using an intention-to-treat analysis. Because the study period was contemporaneous with the COVID-19 pandemic, we accounted for this period-specific event by creating a binary indicator for patients whose follow-up occurred during this time. Models were adjusted for patient age at index, sex, race, residence, neighborhood disadvantage, insurance status, cancer type, cancer stage, Elixhauser Comorbidity Index, pandemic indicator, and enrollment institution.

Exploratory subset analyses comparing RSM to controls planned a priori were conducted by patient race, residence, and neighborhood disadvantage. Ad hoc subset analyses were conducted by insurance type and number of comorbid conditions. For subset analysis, insurance type was categorized as government insurance (Medicare fee-for service, Medicare with supplemental, and Medicaid) or private insurance. Additionally, the Elixhauser Comorbidity Index was categorized as low comorbidity burden (0-1 conditions), moderate comorbidity burden (2-3 conditions), and high comorbidity burden (≥4 conditions). All subset analyses used Firth penalized logistic regression due to smaller sample sizes within strata. For these models, RRs were computed from model estimated proportions, and 95% CIs were computed by inversion of a test statistic for the log(RR). All analyses were performed using SAS software version 9.4 (SAS Institute). Data were analyzed from May to October 2024.

The hospitalization analysis was powered for 80% power (α = .015) to detect a 5% decrease in hospitalizations from an anticipated 6-month hospitalization proportion of 0.25 in the controls, resulting in a recommended population of 2988 patients balanced between groups (odds ratio, 1.33; a small difference). Given the large number of controls, the sample of 5949 (1392 intervention, 4557 controls) was deemed to be sufficient for evaluation. *P* values were 2-sided, and statistical significance was set at P < .05.

## Results

From May 2021 to May 2024, 2242 participants were approached for RSM; 339 patients (15%) declined enrollment in RSM and 511 patients were excluded in the final analysis plan for a final dataset of 1392 RSM patients (median [IQR] age at index date, 61 [51-69] years; 933 [67%] female) (eFigure in Supplement 1). Modest differences between those participating in RSM and those excluded or refused were observed, with the RSM group having higher proportion of patients with breast cancer and Medicaid insurance (eTable in Supplement 1). The proportion of patients enrolled in RSM was consistent with planned program scale-up per year.^24^ The RSM sample included 378 Black patients (27%) and 922 White patients (66%), with 262 patients (19%) living in rural areas and 372 patients (27%) living in areas with high neighborhood disadvantage (Table). Patients receiving RSM were similar to 4557 included controls (median [IQR] age at index date, 62 [53-69] years; 2654 [58%] female), including 1177 Black patients (26%) and 3151 White patients (69%), with 1012 patients (22%) living in rural areas, and 1281 patients (28%) living in areas with high neighborhood disadvantage. Overall, comorbid conditions were similar across both RSM and control groups, with a median (IQR) Elixhauser Comorbidity Index of 3 (2-4). The most common cancer types in both RSM and control groups were breast (28% and 16%), followed by lung (11% and 13%), liver and pancreatic (8% and 9%), and colorectal (7% and 7%). The most common insurance type was private (44% and 40%), followed by Medicare fee-for-service only (33% and 37%) and any Medicaid (11% and 10%).

**Table.** Demographic and Clinical Characteristics of RSM Patients and Historical Controls

Characteristic	Patients, No. (%) (N = 5949)	
	RSM (n = 1392)	Historical (n = 4557)
Race		
Black	378 (27)	1177 (26)
White	922 (66)	3151 (69)
Other or unknown^a^	92 (7)	229 (5)
Sex		
Male	459 (33)	1903 (42)
Female	933 (67)	2654 (58)
Cancer type		
Breast	396 (28)	735 (16)
Cervix	57 (4)	57 (4)
Colorectal	101 (7)	101 (7)
Esophagus	30 (2)	30 (2)
Head and neck	38 (3)	38 (3)
Leukemia	73 (5)	353 (8)
Liver and pancreatic	114 (8)	419 (9)
Lung	160 (11)	580 (13)
Lymphoma	82 (6)	586 (13)
Melanoma	73 (5)	118 (3)
Myeloma	40 (3)	291 (6)
Ovary	57 (4)	280 (6)
Prostate	12 (1)	44 (1)
Urinary system	56 (4)	166 (4)
Uterine	103 (7)	278 (6)
Stage		
Early (I-III)	788 (57)	2256 (50)
Late (IV)	409 (29)	1071 (24)
Hematologic	195 (14)	1230 (27)
Prior treatment	25 (2)	449 (10)
Insurance		
Any Medicaid	153 (11)	474 (10)
Medicare fee-for-service only	456 (33)	1694 (37)
Medicare with supplemental	88 (6)	232 (5)
Private	612 (44)	1816 (40)
Other/none	83 (6)	341 (7)
Highest neighborhood disadvantage	372 (27)	1281 (28)
Patient residence		
Rural	262 (19)	1012 (22)
Urban	1130 (81)	3545 (78)
Elixhauser Comorbidity Index score, median (IQR)	3 (2-4)	3 (2-4)
Low burden (0-1)	289 (21)	1129 (25)
Moderate burden (2-3)	595 (43)	1832 (40)
High burden (≥4)	508 (36)	1596 (35)
Age at index date, median (IQR), y^b^	61 (51-69)	62 (53-69)

Abbreviation: RSM, remote symptom monitoring.

^a^ Includes American Indian or Alaska Native, Asian, Hispanic or Latino, Native Hawaiian or Pacific Islander, and other.

^b^ Index date is the RSM start date for intervention patients or the date of the first RSM qualifying treatment for controls.

**Figure 1.**
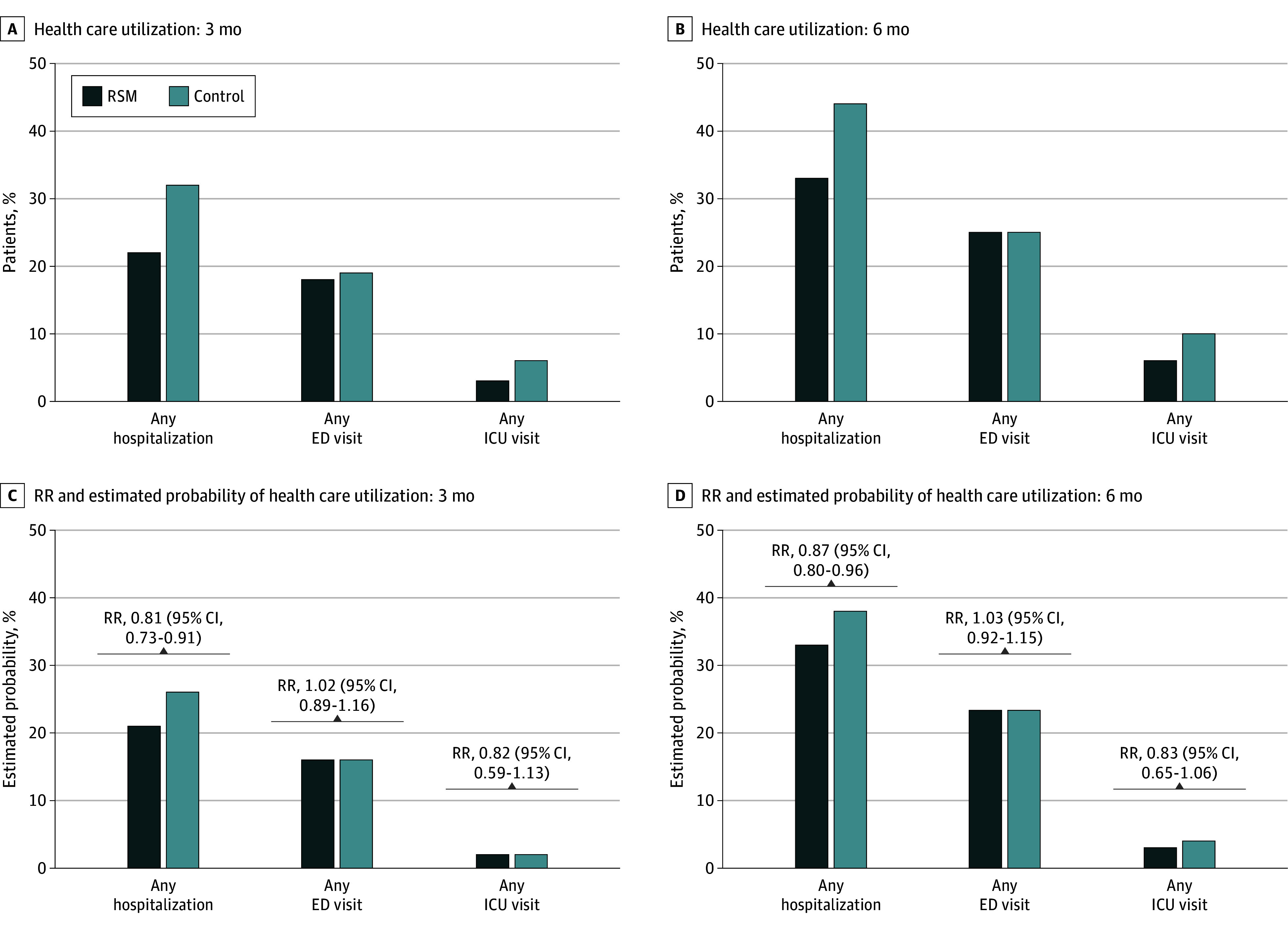
Unadjusted Frequency and Adjusted Relative Risk (RR) of Health Care Utilization at 3 and 6 Months ED indicates emergency department; ICU, intensive care unit; and RSM, remote symptom monitoring.

The unadjusted proportions of patients with a hospitalization were lower among patients receiving RSM compared with control patients at 3 months (22% vs 32%) and 6 months (33% vs 44%) (Figure 1A). In adjusted analysis, risk of hospitalization was 19% lower at 3 months (RR, 0.81; 95% CI, 0.73-0.91) and 13% lower at 6 months (RR, 0.87; 95% CI, 0.80-0.96) for patients receiving RSM vs controls (Figure 1B). Risk of ICU admission was not statistically significant among the RSM vs control populations (3 months: RR, 0.82; 95% CI, 0.59-1.13; 6 months: RR, 0.83; 95% CI, 0.65-1.06). ED visits were similar in both groups (3 months: RR, 1.02; 95% CI, 0.89-1.16; RR, 1.03; 95% CI, 0.92-1.15).

**Figure 2.**
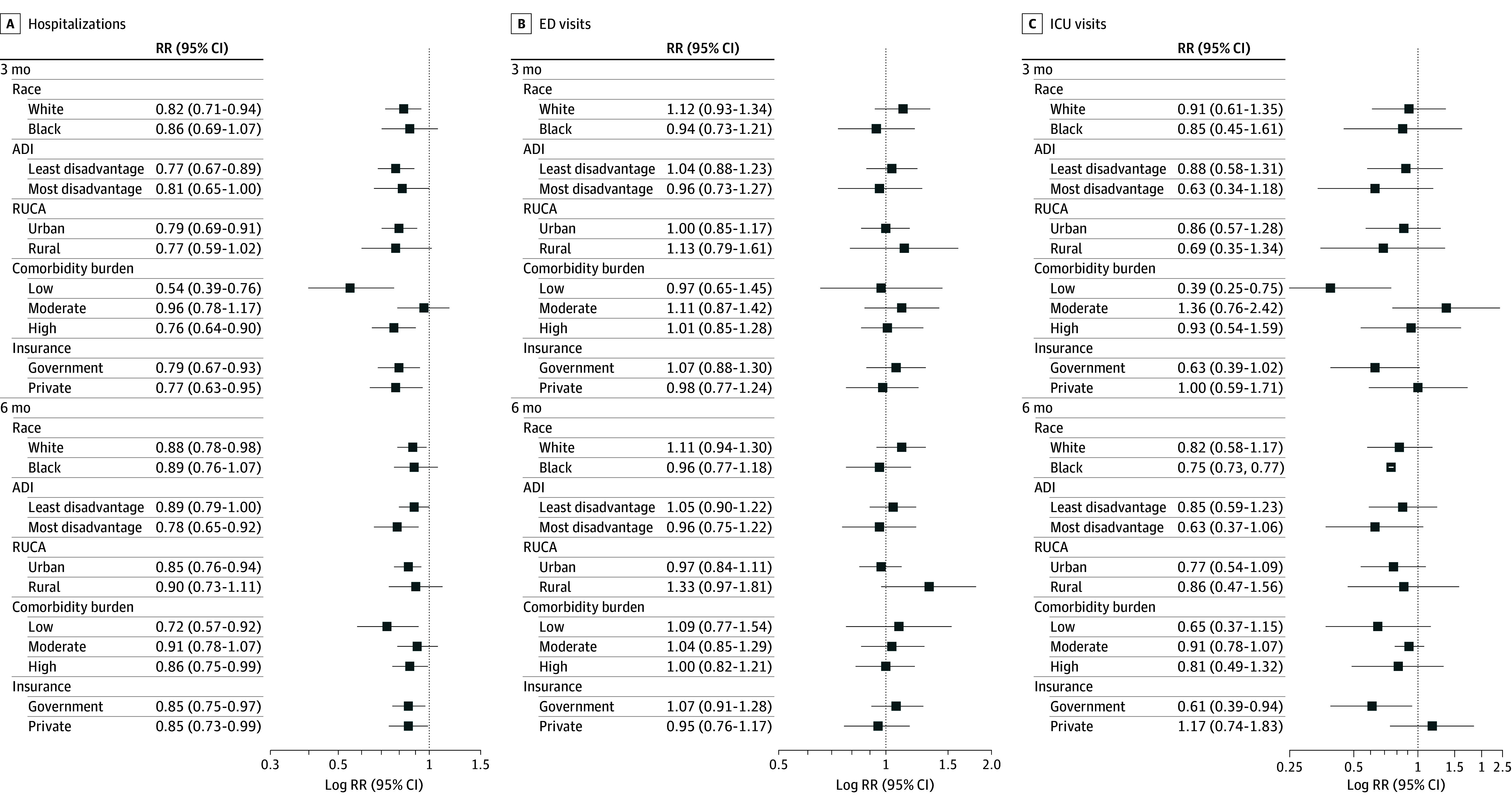
Comparison of Health Care Utilization Among Patients Receiving Remote Symptom Monitoring vs Control Patients by Subset ADI indicates Area Deprivation Index; ED indicates emergency department; ICU, intensive care unit; and RUCA, Rural-Urban Commuting Area.

Similar patterns in RR were observed for hospitalizations (Figure 2A), ED visits (Figure 2B), and ICU admissions (Figure 2C) across all subset analysis at 3 months and 6 months. When stratified, we did not find statistically significant differences in risk of hospitalization associated with RSM for Black patients (3 months: RR, 0.86; 95% CI, 0.69-1.07; 6 months: RR, 0.89; 95% CI, 0.76-1.07), patients residing rurally (3 months: RR, 0.77; 95% CI, 0.59-1.02; 6 months: RR, 0.90; 95% CI, 0.73-1.11), and patients living in areas of high neighborhood disadvantage (3 months: RR, 0.77; 95% CI, 0.59-1.02; 6 months: RR, 0.78; 95% CI, 0.65-0.92) compared with historical controls. However, patients receiving RSM with a high comorbidity burden (≥4) had a lower risk of hospitalizations than historical controls (3 months: RR, 0.76; 95% CI, 0.64-0.90; 6 months: RR, 0.86; 95% CI, 0.75-0.99). Similar results were seen for RSM patients with low comorbidity burden (0-1). Ad hoc analysis by insurance type also demonstrated similar results.

## Discussion

This large scale, EMR-integrated nonrandomized controlled trial found that ePRO-based RSM was associated with decreased hospitalizations across a diverse population of patients with cancer receiving active treatment compared with historical controls. This finding expands to the clinical setting the benefits associated with RSM for health care utilization from several previous randomized clinical and nonrandomized trials in which favorable impacts of similar programs were observed on health care utilization.^7,9,10,11,15,29,30,31^ Importantly, this analysis suggested benefit was observed across patient populations with respect to race, rurality, and socioeconomic status. As these benefits are observed for all groups, it will be imperative to ensure engagement of diverse populations in future efforts as use of ePROs and RSM to minimize disparities. Given our previous work showing Black patients had a 3-fold higher likelihood of declining RSM participation and those living in areas of high area deprivation were less likely to complete RSM surveys,^17^ attention to equity should be a key focus of future implementation efforts.

This program showed favorable benefit for participants regardless of insurance status, a finding that is also critical when considering application of payment reform in the US. In oncology, the EOM only includes patients with primary Medicare insurance.^6^ Few other payers have programs available to support the infrastructure needed for ePROs, which results in a small subset of the overall population of patients with cancer with a reimbursement strategy for capture of ePROs. There is a tangible need for other payers, including both private payers and Medicaid, to consider how to best support value-oriented practice transformation activities, such as ePRO-based RSM. This need is pressing, as EOM practices must make difficult choices about whether to deploy RSM for a Medicare population only or to absorb both the cost of infrastructure and the loss of revenue from downstream reductions in health care utilization.

While this study focused specifically on an oncology population, more than one-third of these patients had 4 or more secondary comorbid conditions, who also benefited from RSM with a 24% reduction in relative risk at 3 months. This finding suggests the possibility that ePRO-based RSM may be beneficial in other subspecialties, particularly if coupled with other remote monitoring practices. In a heart failure meta-analysis, home monitoring using implantable devices to track hemodynamic parameters was associated with decreased hospitalization and mortality rates compared with standard monitoring.^32^ While further research will be needed to integrate the multiple approaches to remote monitoring for patients with chronic disease, this study may serve as a model for scalable, EMR-integrated care and patient-centered care delivery in other disease conditions.

### Limitations

This study has some limitations. We recognize that not all patients participated in the RSM program, both due to incomplete roll-out of the program and patients declining to enroll. This may result in selection bias that may not be fully controlled for within our models due to unmeasured confounding. However, using a pragmatic design to evaluate standard-of-care implementation without requiring consent allows for greater participation of vulnerable populations and allows for evaluation in clinical settings that complements existing numerous trials demonstrating benefit of ePROs for patients.^7,9,10,11,15,30,31^ Although implementation within cancer centers in Alabama offers a population with low socioeconomic status and a high proportion of Black patients, there were few (<5%) Hispanic patients and other subpopulations in our cohort who may have unique language and cultural needs.^33^ We also recognize that the exploratory, preplanned subgroup analysis has limited power to detect statistically significant differences; thus, these analyses are hypothesis generating and require further testing in future studies. Furthermore, these centers represent a large National Cancer Institute–designated comprehensive cancer center, and a smaller academic cancer center and may not fully capture the experience of patients within a community practice setting. However, we are optimistic, in that our findings were consistent with the evaluation conducted by Patt et al^15^ within a community practice. We note limitations of EMR-based analyses in terms of data accuracy and quality, particularly because utilization outside the participating centers would not be captured within the EMR. We also did not assess outcomes for patients who declined or had incomplete survey participation, which could impact the overall utilization for key populations (eg, Black patients, those in areas with greater socioeconomic disadvantage) that are known to have lower engagement.^17^ In addition, our primary analysis lacks a full assessment of outcomes that occur outside of the participating health systems. Furthermore, our analysis spans the timeframe of the COVID-19 pandemic. Although we accounted for this within our models, we cannot rule out the potential for unmeasured confounding affecting our results.

## Conclusions

In a large scale, clinical practice population, RSM using ePROs was associated reduced health care utilization in the form of hospital admission in a diverse oncology population. Findings were observed in a diverse patient population, supporting broad applicability and highlighting the potential for this EOM-required practice transformation activity to impact value in cancer care delivery.

## Notes

Supplement 1. **eFigure.** Exclusion Cascade to Determine Final RSM Analytic Cohort **eTable.** Demographic and Clinical Characteristics by Analytic Cohort, Patients Excluded, and Patients Declined

Supplement 2.
Data Sharing Statement
