# Supplementary material for: Remote Symptom Monitoring With Electronic Patient-Reported Outcomes in Clinical Cancer Populations
Source: JAMA Netw Open. 2025 May 13;8(5):e259852. doi: 10.1001/jamanetworkopen.2025.9852 (PMC12076171; doi:10.1001/jamanetworkopen.2025.9852)
Supplement: Supplement 1. — eFigure. Exclusion Cascade to Determine Final RSM Analytic Cohort eTable. Demographic and Clinical Characteristics by Analytic Cohort, Patients Excluded, and Patients Declined [file jamanetwopen-e259852-s001.pdf]

Supplemental Online Content

Rocque GB, Franks JA, Deng L, et al. Remote symptom monitoring with electronic patient-reported outcomes in clinical cancer populations. *JAMA Netw Open*. 2025;8(5):e259852. doi:10.1001/jamanetworkopen.2025.9852

eFigure. Exclusion Cascade to Determine Final RSM Analytic Cohort

eTable. Demographic and Clinical Characteristics by Analytic Cohort, Patients Excluded, and Patients Declined

This supplemental material has been provided by the authors to give readers additional information about their work.

© 2025 Rocque GB et al. *JAMA Network Open*.

Supplemental Figure 1: Exclusion Cascade to determine final RSM analytic cohort

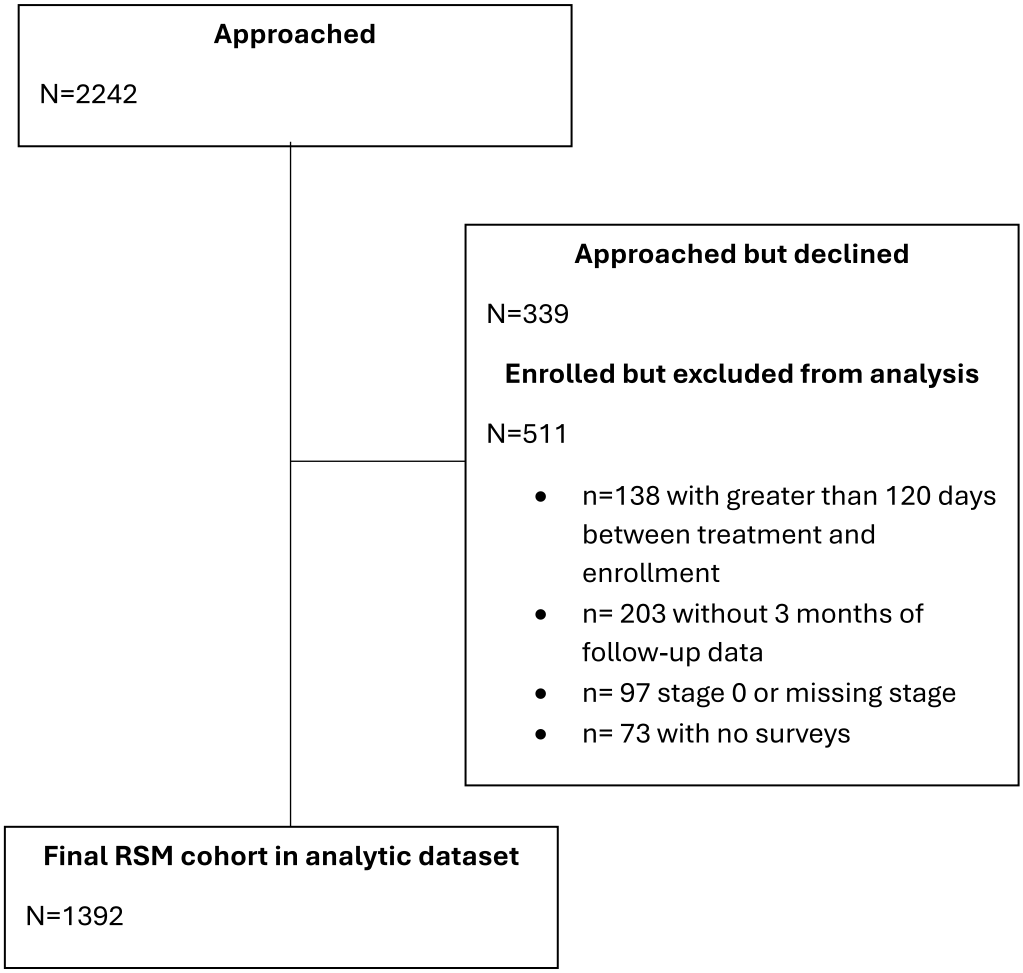

© 2025 Rocque GB et al. *JAMA Network Open*.

Supplemental Table 1. Demographic and clinical characteristics by analytic cohort, patients excluded, and patients declined (N=2242)

	**Analytic sample**<br><br>**n=1392**<br>**n(%)**	**Enrolled but Excluded**<br>**n=511**<br>**n(%)**	**Approached but declined**<br>**n=339**<br>**n(%)**
**Race**			
White	922 (66)	338 (66)	210 (62)
Black	378 (27)	136 (27)	96 (28)
[b]Other or unknown	92 (7)	37 (7)	33 (10)
**Sex**			
Male	459 (33)	185 (36)	167 (49)
Female	933 (67)	326 (64)	172 (51)
**Cancer type**			
Breast	396 (28)	121 (24)	37 (11)
Cervix	57 (4)	19 (4)	7 (2)
Colorectal	101 (7)	31 (6)	45 (13)
Esophagus	30 (2)	5 (1)	10 (3)
Head and neck	38 (3)	20 (4)	12 (4)
Leukemia	73 (5)	25 (5)	30 (9)
Liver and pancreatic	114 (8)	47 (9)	47 (14)
Lung	160 (11)	45 (9)	39 (12)
Lymphoma	83 (6)	43 (8)	22 (6)
Melanoma	73 (5)	20 (4)	14 (4)
Myeloma	40 (3)	52 (10)	22 (6)
Ovary	57 (4)	32 (6)	9 (3)
Prostate	12 (1)	5 (1)	5 (2)
Urinary system	56 (4)	10 (2)	19 (6)
Uterine	103 (7)	36 (7)	21 (6)
**Insurance**			
Any Medicaid	153 (11)	38 (7)	18 (5)
Medicare fee-for-service only	546 (33)	206 (40)	160 (47)
Medicare with supplemental	88 (6)	20 (4)	22 (6)
Private	612 (44)	214 (42)	109 (32)
Other/none	83 (6)	33 (6)	30 (9)
**Neighborhood Disadvantage (Area Deprivation Index- ADI)**			
Most disadvantaged	372 (27)	360 (70)	241 (71)
Least disadvantaged	1020 (73)	151 (30)	98 (29)
**Patient Residence (Rural-Urban Communing Area-RUCA)**			
Rural	262 (19)	105 (21)	73 (22)
Urban	1130 (81)	405 (79)	266 (78)
**[a]Age at index [Median,(IQR)]**	61 (51-69)	62 (52-69)	67 (58-73)
*N=2263 eligible to be approached, n=21 omitted due to pending status at time of analysis			

© 2025 Rocque GB et al. *JAMA Network Open*.
