# Supplementary material for: Remote Symptom Monitoring With Electronic Patient-Reported Outcomes in Clinical Cancer Populations
Source: JAMA Netw Open. 2025 May 13;8(5):e259852. doi: 10.1001/jamanetworkopen.2025.9852 (PMC12076171; doi:10.1001/jamanetworkopen.2025.9852)
Supplement: Supplement 2. — Data Sharing Statement [file jamanetwopen-e259852-s002.pdf]

Data Sharing Statement

Rocque. Remote Symptom Monitoring With Electronic Patient-Reported Outcomes in Clinical Cancer Populations. *JAMA Netw Open*. Published May 13, 2025.
doi:10.1001/jamanetworkopen.2025.9852

Data
Data available: Yes
Data types: Deidentified participant data, Data dictionary
How to access data: Data will be made upon request of PI with appropriate permissions and support.
When available: beginning date: 01-01-2027

Supporting Documents
Document types: Statistical/analytic code
How to access documents: This will be made available by request of the PI.
When available: beginning date: 01-01-2027

Additional Information
Who can access the data: This will be made available to researchers whose proposed use of the data has been approved.
Types of analyses: Evaluation of remote symptom monitoring.
Mechanisms of data availability: with investigator support after approval of the proposal with a signed data access agreement.
